# Effects of lidocaine-plus-meloxicam treatment on behavioral and physiological changes, and leukocyte heat shock protein 90 gene expression after surgical castration in Hanwoo bulls

**DOI:** 10.3389/fvets.2024.1465844

**Published:** 2024-12-04

**Authors:** Ingu Cho, Seonpil Yoo, Da Jin Sol Jung, Jaesung Lee, Seok-Hyeon Baek, Sang Yeob Kim, Jinoh Lee, Dohyun Kim, Hyun Jin Kim, Myunggi Baik

**Affiliations:** ^1^Department of Agricultural Biotechnology and Research Institute of Agriculture and Life Sciences, College of Agriculture and Life Sciences, Seoul National University, Seoul, Republic of Korea; ^2^Animal Welfare Program, Faculty of Land and Food Systems, The University of British Columbia, Vancouver, BC, Canada; ^3^Institutes of Green Bio Science Technology, Seoul National University, Pyeongchang-gun, Gangwon, Republic of Korea

**Keywords:** welfare, stress, bull, non-steroidal anti-inflammatory drug, local anesthetic

## Abstract

This study examined the effects of surgical castration and lidocaine-plus-meloxicam treatment on growth, physiology, behaviors, and leukocyte heat shock protein 90 (HSP 90) gene expression in Hanwoo (Korean cattle) bulls. Twenty Hanwoo bulls (body weight 248.8 ± 28.5 kg, age 9.4 ± 1.04 months) were assigned to three treatments: surgical castration with lidocaine injection (5 mL in the scrotum) and oral meloxicam administration (1 mg/kg body weight; LM; *n* = 7); surgical castration with placebo injection (5 mL of 0.9% NaCl) and oral placebo administration (lactose, 1 mg/kg body weight; CAS; *n* = 7); and shame castration (SHAM; *n* = 6). Meloxicam and lactose were administered 3 h before castration, and lidocaine and NaCl were injected immediately before castration. Surgical castration was performed with a Newberry knife and a Henderson castration tool. Wight was measured the day before and 14 d after castration, and behavior was observed from 0.5 h to 4.5 h post-castration. Blood was collected at −1 d, 0.5 h, 6 h, 1 d, 3 d, 7 d, and 14 d after castration to measure plasma cortisol, haptoglobin, and leukocyte HSP 90 mRNA. Castration tended to decrease average daily gain (*p* = 0.06), but the LM treatment did not affect weight gain. Bulls in CAS showed higher cortisol concentration (*p* < 0.05) at 0.5 and 6 h after castration compared to SHAM, with no difference between CAS and LM. Castration increased haptoglobin concentration at 1 and 3 d after castration (*p* < 0.05) while LM bulls showed decreased haptoglobin concentration (*p* < 0.05) than CAS bulls at these times. HSP90 mRNA was increased at 6 h post-castration while LM did not reduce its expression, suggesting HSP90 may serve as an acute stress marker in castrated bulls. Castration increased (*p* < 0.05) frequencies of drinking, lying, walking, leg lifting, kicking, and stiff gait, while decreasing (*p* < 0.05) eating frequency. LM alleviated (*p* < 0.05) drinking, leg lifting, kicking, and stiff gait. Collectively, castration resulted in physiological changes, increased leukocyte HSP90 gene expression, and altered behaviors. These findings suggest that lidocaine-plus-meloxicam treatment partially mitigates pain and inflammation in the castrated bulls.

## 1 Introduction

Castration is a common management practice conducted in beef industry to reduce aggression temperament, to prevent unwanted breeding, and to improve meat quality (1, 2). However, castration is known to cause stress and pain, supported by inflammatory indicators, behavioral and physiological changes (3, 4). Previous studies have demonstrated that combination of a local anesthetic and a non-steroidal anti-inflammatory drug (NSAID) can effectively relieve pain associated with castration, as the local anesthetic reduces pain during castration and the NSAID reduces pain afterward (5). For example, a combination of lidocaine and flunixin reduced pain following surgical castration, as evidenced by decreased plasma cortisol level and haptoglobin level (6). Additionally, both surgically and band-castrated calves exhibited increased standing time and a high number of lying bouts compared to control calves, whereas calves treated with meloxicam showed no such difference (3). Within local anesthetics, lidocaine has been commonly used while bupivacaine has been tested with liposome suspension (7) or as a topical anesthetic (8, 9). Lidocaine injection is beneficial not only during the procedure but also for hours afterward, considering the anesthetic effect lasts for 180 min (10). So far, however, there has been no behavioral evidence of a lasting effect of lidocaine for hours after castration. A study that assessed the effect of lidocaine treatment on pain after Burdizzo castration failed to find significant behavioral changes for 6 h, based on a scan sampling method (11). Most studies evaluating the combined effect of a NSAID and local anesthesia have used lidocaine and ketoprofen (5). The combined effect of the local anesthesia and flunixin was assessed, but found limited pain relief after surgical castration (6). Given that meloxicam has a longer half-life than other NSAIDs (12), the combination of meloxicam and local anesthesia may be more effective in relieving pain after castration. Several studies examined the effect of the combination of lidocaine and meloxicam on pain related to surgical castration (13, 14). However, the impact of this combination on behavior for hours after surgical castration has not been investigated well.

Advances in molecular biology have enabled the early diagnosis of diseases through gene expression profiling in humans (15, 16). However, gene expression has yet to be utilized as a diagnostic tool in cattle. Heat shock proteins (HSPs) are molecular chaperones that play a crucial role in cellular stress responses (17), including the modulation of pain mechanisms. Recent research has highlighted their involvement in pain regulation, suggesting that targeting HSPs could offer new avenues for pain relief in both humans and animals (18). However, there is a lack of literature regarding the impact of castration pain on HSP gene expression in bulls.

The aim of this study was to investigate the effects of treatment of lidocaine with meloxicam on behavioral and physiological changes, as well as leukocyte HSP 90 gene expression, following surgical castration of Hanwoo (Korean cattle) bulls.

## 2 Materials and methods

### 2.1 Animals, housing, and diet

Twenty healthy Hanwoo bulls were purchased at auction and acclimated for 2 weeks prior to castration. During this period, each bull was individually provided with a diet consisting of a commercial concentrate (1.6% of body weight), 3 kg of timothy, and 1 kg of alfalfa twice daily at 0800 and 1,600 h. Bulls were tethered to stanchions during feeding and feed intake was monitored. All bulls consistently consumed more than 80% of their feed daily, indicating no health issue, so all bulls were included in the experiment. The animals were fed the same feeds continuously throughout the experiment. The intakes of timothy hay, alfalfa hay, and concentrate were determined by subtracting the weight of leftovers from the initially provided feed weights. The ingredients of the concentrate and the chemical composition of the feed were provided in Supplementary Table 1. Water was provided freely.

### 2.2 Treatments

One day (d) prior to castration (−1 d), 20 bulls (mean age 9.4 ± 1.04 months; mean weight 248.8 ± 28.5 kg) were weighed. In South Korea, calves are typically weaned at 3 months, transported to a new farm at 6 months, and castrated between 6 to 9 months of age. We followed this conventional castration timing. Bulls were randomly allocated to one of three treatments: castration with saline solution injection and oral lactose administration (CAS; *n* = 7); castration with local anesthetic injection and oral meloxicam administration (LM; *n* = 7); or SHAM castration with no treatment (SHAM; *n* = 6). During the castration procedure, bulls were haltered and restrained in a squeeze chute for approximately 5 min. Bulls in the LM group received meloxicam (oral, 1 mg/kg BW) 3 h before castration and 5 mL of 2% lidocaine hydrochloride at the neck of the scrotum subcutaneously 10 min before castration. Ten minutes after lidocaine injection, surgical castration was performed with a Newberry knife to open the distal part of the scrotum, followed by the Henderson castration tool to twist the exposed spermatic cord, as described (11). Immediately after the removal of the testicles, 15 mL of vitamin K3 (IM, 20 mg/mL menadione sodium bisulphite trihydrate, Samyang Anipharm, Seoul, Korea) were injected into the neck for hemostasis, and the surgical site was coated with aluminum powder (Vetoquinol Korea, Gyeonggi, Korea) to prevent bleeding and infection. In the CAS group, the castration procedure was the same as the LM group, except that bulls received a 5 mL of 0.9% Nacl solution and lactose (1 mg/kg body weight) instead of lidocaine and meloxicam. Bulls in the SHAM group were haltered and restrained in a squeeze chute for 5 min without undergoing castration. Body weight was measured again at the end of the experiment (14 d).

### 2.3 Blood collection and analysis

Two 10-mL blood samples were collected in ethylenediaminetetraacetic acid (EDTA)-treated vacutainers (BD Biosciences, San Jose, CA, United States) by jugular venepuncture 1 d before castration (−1 d) and 0.5 and 6 h, and 1, 3, 7, and 14 d after castration, then used to prepare blood plasma and isolate leukocytes. Blood samples were kept in an ice box during sampling. Plasma was separated by centrifugation at 1500 x g at 4°C for 15 min and stored at −70°C until use in enzyme-linked immunosorbent assays (ELISAs).

Leukocytes were isolated from blood as described by O’Loughlin et al. (19). Briefly, blood from a vacutainer tube was mixed into hypotonic solution for red blood cell lysis. The leukocytes were restored in a hypertonic solution and then collected as pellets after centrifugation. Leukocyte pellets were suspended in 1 mL of TRI Reagent (Sigma-Aldrich Ireland, Dublin, Ireland) and stored in sterile tubes at −70°C.

Plasma cortisol was analyzed using a salivary cortisol enzyme immunoassay kit (Salimetrics, State College, PA, United States). The respective intra-and inter-assay coefficients of variance of the cortisol kit were 4.5 and 4.9%. Plasma haptoglobin was analyzed using a bovine haptoglobin ELISA kit (Genway Biotech, San Diego, CA, United States). The intra-and inter-assay coefficients of variance of the haptoglobin kit were 6.1 and 5.8%, respectively. Previously, we validated all of the analytical methods (4, 6).

### 2.4 RNA extraction and quantitative reverse transcription polymerase chain reaction

Total RNA was isolated from leukocytes using TRI Reagent, in accordance with the manufacturer’s protocol. RNA was quantified using a NanoPhotometer (Implen, Munich, Germany), and quality was checked by agarose gel electrophoresis with ethidium bromide staining of the 28S and 18S bands and a Bioanalyzer (Agilent Technologies, Santa Clara, CA, United States), as previously described (4). Total RNA was synthesized into cDNA using the iScript cDNA synthesis kit (Bio-Rad, Hercules, CA, United States), in accordance with the manufacturer’s instructions. We used 18S rRNA as a reference gene because expression of 18S rRNA was more uniform compared to *β*-actin, and the ribosomal protein lateral stalk subunit P0 in the leukocytes. Primer sequences, which span an exon-exon junction and melting temperature ranged from 59.1°C to 61.0°C, were identified using Primer-BLAST at NCBI. Supplementary Table 2 lists the primers used. qPCR was performed using QuantiTect SYBR Green RT-PCR Master Mix (QIAGEN, Hilden, Germany), as previously described (20). We followed the “Minimum Information for Publication of Quantitative Real-Time PCR Experiments” guidelines for qPCR (21) including qPCR efficiency calculation. All qPCR analyses were conducted in a 25 μL total reaction volume that contained 20 ng cDNA, 12.5 μL SYBR Green RT-PCR Master Mix, and 1.25 μL of 10 μM primers. The thermal cycling parameters were 95°C for 15 min, followed by 40 cycles at 94°C for 15 s, 55°C for 30 s, and 72°C for 30 s. An annealing temperature of 55°C was used for amplification of all genes, resulting in a single major peak in all cases. The ΔΔCT method (22) was used to determine the relative fold changes in gene expression. Primer amplification efficiency was tested using serial diluted cDNA, as described (21). Briefly, the log-transformed concentration of the cDNA template was plotted on the *x*-axis and the quantification cycle (Cq) was plotted on the *y*-axis. PCR efficiency was determined from the slope of this plot (PCR efficiency = 10^–1/slope^ – 1). Primer efficiencies for 18 s ribosomal RNA and heat shock protein 90 genes were 98 and 97%, respectively, which is acceptable for qPCR (Supplementary Table 2).

### 2.5 Behavior analysis

When the animals were restrained for treatment, they were labeled with colored duct tape. Animal behavior was recorded using smartphones (Galaxy S10, Samsung, Gyeonggi, Korea) for 4 h, starting from 0.5 h to 4.5 h after castration. From the 4 h video clips, behaviors including eating, drinking, lying, walking, standing, leg lifting, kicking, lesion licking, head turn, stiff gait, scratch, head shake, and head paw were recorded. These behavaiors were defined based on published research on surgical castration (9, 23, 24) and described in detail in Supplementary Table 3.

### 2.6 Statistical analysis

Before the analyses, the data distribution was checked using the UNIVARIATE procedure of SAS. Data with non-normal distributions were logarithmically transformed. Blood data were analyzed using the repeated-measures MIXED procedure of SAS (SAS Institute, Cary, NC, United States). The experimental unit was an individual bull. The statistical model included the fixed effects of treatment and time, as well as the treatment × time interaction. Bulls were considered random effects. Three covariance structures (auto-regressive type 1, compound symmetry, and Toeplitz) were tested, and the covariance structure with the lowest Schwarz’s Bayesian information criterion was chosen. The Tukey–Kramer test was used for multiple comparisons. The initial BW was used as a covariate for the final weight. Growth performance and behavior data were analyzed using one-way analysis of variance. The threshold for statistical significance was set to *p* < 0.05; trends were declared at 0.05 ≤ *p* ≤ 0.10.

## 3 Results

### 3.1 Growth performance

No treatment effects were detected (*p* > 0.10) on final BW and the roughage and concentrate intakes (Table 1). However, the average daily gain (ADG) and feed efficiency tended to be lower (*p* = 0.06 and *p* = 0.07, respectively) in CAS and LM (i.e., CAS and LM groups) than in SHAM (i.e., SHAM group).

**Table 1:** Effects of castration and lidocaine-plus-meloxicam (LM) treatment on growth performance of Hanwoo bulls.

Item	Treatment^1^			SEM^2^	*p*-value
	SHAM	CAS	LM		
Body weight (BW), kg					
Initial BW (−1 d), kg	248	249	249	6.4	1.00
Final BW (14 d), kg	263	256	257	5.7	0.89
Daily timothy intake (kg DM/d)	2.59	2.65	2.64	0.013	0.21
Daily alfalfa intake (kg DM/d)	0.73	0.80	0.80	0.022	0.32
Daily concentrate intake (kg DM/d)	3.34	3.53	3.49	0.083	0.64
Total feed intake (kg DM/d)	6.65	6.97	6.94	0.104	0.42
Average daily gain (kg/d from −1 d to 14 d)	1.02^A^	0.52^B^	0.54^B^	0.098	0.06
Feed efficiency (kg of gain/kg of feed)	0.15^A^	0.08^B^	0.08^B^	0.015	0.07

A Means within a same row with different superscripts differ at *p* < 0.10.

B Means within a same row with different superscripts differ at *p* < 0.10.

1 SHAM, no castration with no treatment; CAS, castration with NaCl injection and administering oral lactose; LM, castration with lidocaine hydrochloride injection and administering oral meloxicam.

2 Standard error of mean.

### 3.2 Plasma stress and inflammation indicators

**Figure 1:**
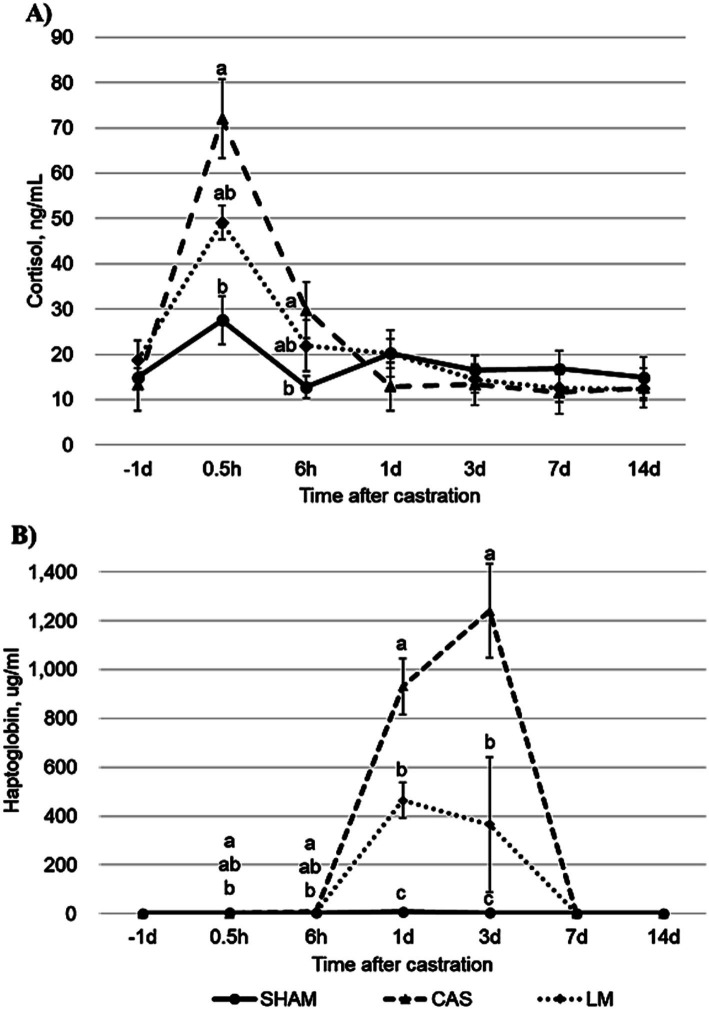
Effects of castration and lidocaine plus meloxicam on the plasma (A) cortisol and (B) haptoglobin concentrations of Hanwoo bulls (SHAM, no castration with no treatment; CAS, castration with NaCl injection and oral lactose; LM, castration with lidocaine hydrochloride injection and oral meloxicam). Values are presented as means + standard errors (SEs; *n* = 6 or 7/treatment). a–c Means with differing superscripts differ (*p* < 0.05) at each time point (Tukey–Kramer test).

No treatment effect was detected (*p* = 0.96) for the plasma cortisol concentration (Figure 1A). However, a treatment × time interaction was detected for plasma cortisol (*p* = 0.01). At 0.5 and 6 h after castration, the cortisol concentration was higher (*p* < 0.05) in CAS than in SHAM; however, no differences were observed (*p* > 0.10) between the two groups (SHAM and CAS) and LM (Figure 1A). Treatment, time, and interaction effects were detected (all *p* < 0.01) for haptoglobin (Figure 1B). At 1 and 3 d after castration, CAS had a higher (*p* < 0.05) haptoglobin concentration than LM and SHAM; it was higher in LM (*p* < 0.05) than in SHAM (Figure 1B).

### 3.3 Leukocyte heat shock protein 90 (HSP90) expression

**Figure 2:**
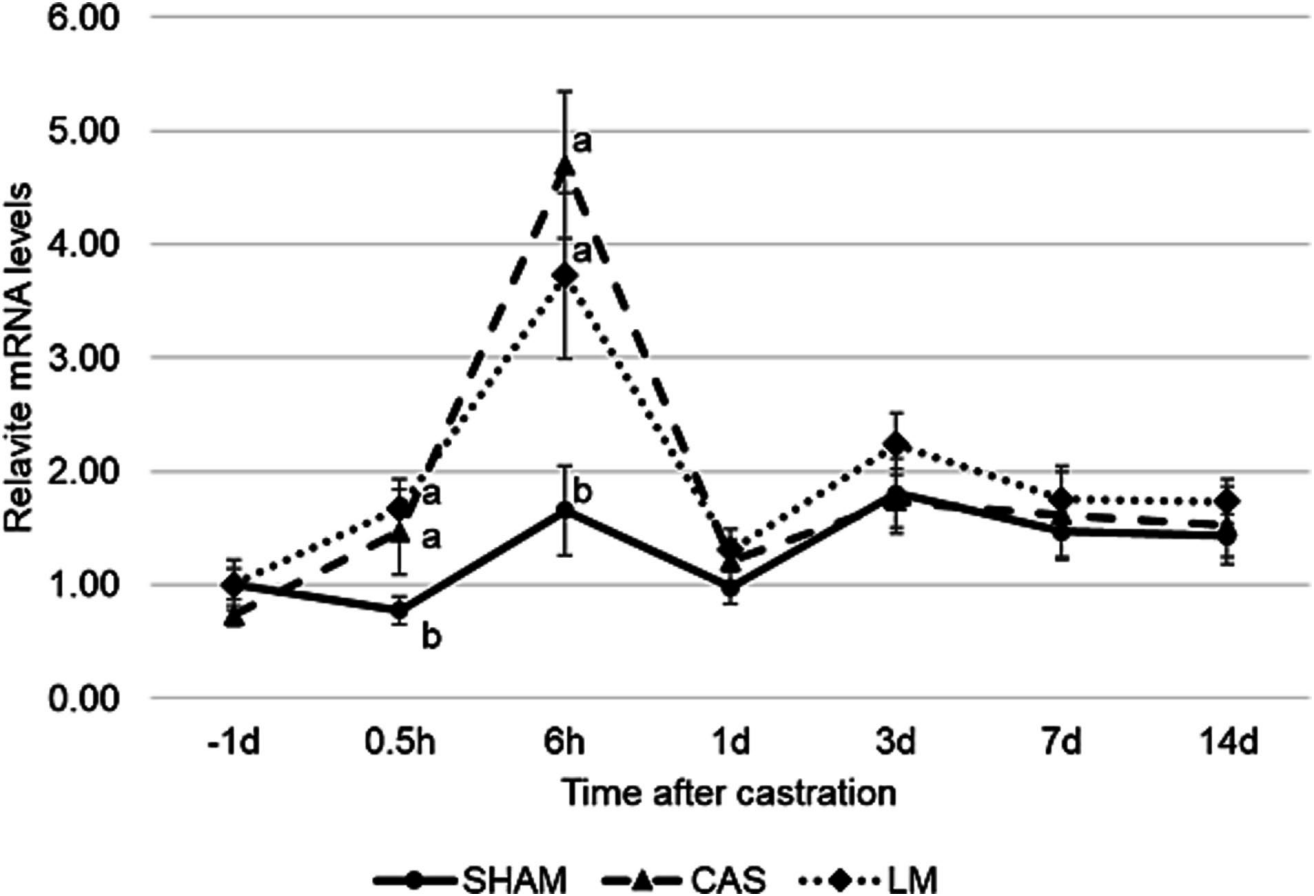
Effects of castration and lidocaine plus meloxicam on the blood leukocyte heat shock protein 90 (HSP90) mRNA level of Hanwoo bulls (SHAM, no castration with no treatment; CAS, castration with NaCl injection and oral lactose; LM, castration with lidocaine hydrochloride injection and oral meloxicam). mRNA levels were determined using qPCR and normalized to 18S ribosomal RNA. mRNA levels 1 d before castration in SHAM were normalized to 1.0. Values are presented as means + SEs (*n* = 6 or 7/treatment). a, b Means with differing superscripts differ (*p* < 0.05) among treatment groups at each time point (Tukey–Kramer test).

A treatment effect was observed (*p* = 0.04) for HSP90. A treatment × time interaction was observed (*p* = 0.04). LM had higher (*p* < 0.05) HSP90 mRNA levels than SHAM; however, no differences were observed (*p* > 0.10) between the two groups (SHAM and LM) and CAS. At 0.5 and 6 h after castration, the HSP90 mRNA level was higher (*p* < 0.05) in CAS and LM than in SHAM (Figure 2).

### 3.4 Behavior observations

Eating duration and frequency were greater (*p* < 0.01) in SHAM (42.9 min/4 h and 36.8/4 h) than in CAS (19.9 min/4 h and 13.4/4 h) and LM (28.3 min/4 h and 19.6/4 h; Table 2). Drinking frequency was greater (*p* = 0.01) in CAS (14.7/4 h) than in SHAM (4.7/4 h) and LM (4.6/4 h). Drinking duration tended to be longer (*p* < 0.10) in CAS (10.4 min/4 h) than in SHAM (2.7 min/4 h) and LM (4.0 min/4 h).

**Table 2:** Effects of castration and lidocaine-plus-meloxicam (LM) treatment on behaviors of Hanwoo bulls.

	Treatment^1^			SEM^2^	*p*-value
	SHAM	CAS	LM		
Eating frequency (no./4 h)	36.8^a^	13.4^b^	19.6^b^	3.31	<0.01
Eating duration (min/4 h)	42.9^a^	19.9^b^	28.3^b^	3.02	<0.01
Eating duration/frequency (min/no.)	1.4	1.6	1.6	0.15	0.77
Drinking frequency (no./4 h)	4.7^b^	14.7^a^	4.6^b^	1.76	0.01
Drinking duration (min/4 h)	2.7^B^	10.4^A^	4.0^B^	1.42	0.05
Drinking duration/frequency (min/no.)	0.5	0.6	0.7	0.10	0.79
Lying frequency (no./4 h)	4.8^b^	17.1^a^	12.0^a^^,^^b^	1.74	<0.01
Lying duration (min/4 h)	89.3	59.3	57.9	7.84	0.20
Lying duration/frequency (min/no.)	19.5^a^	3.8^b^	4.9^b^	1.78	<0.01
Walking frequency (no./4 h)	209^b^	620^a^	529^a^	53.7	<0.01
Walking duration (min/4 h)	25.4^b^	51.5^a^	46.0^a^	3.79	<0.01
Walking duration/frequency (min/no.)	0.13^a^	0.08^b^	0.09^b^	0.008	0.04
Standing duration (min/4 h)	125	129	136	5.7	0.76
Leg lifting (no./4 h)	23^c^	311^a^	159^b^	31.7	<0.01
Kicking (no./4 h)	16.5^b^	83.3^a^	40.6^b^	7.59	<0.01
Lesion licking (no./4 h)	14.0	30.6	11.6	4.31	0.14
Head turn (no./4 h)	48	103	57	13.3	0.19
Stiff gait (no./4 h)	1.3^c^	93.9^a^	24.9^b^	9.52	<0.01
Scratch (no./4 h)	49.8^a^	35.3^b^	22.1^c^	2.96	<0.01
Head shake (no./4 h)	26.7	40.1	74.6	19.94	0.63
Head paw (no./4 h)	3.3	1.4	1.4	0.45	0.15

a Means within a same row with different superscripts differ at *p* < 0.05.

b Means within a same row with different superscripts differ at *p* < 0.05.

A Means within a same row with different superscripts differ at *p* < 0.10.

B Means within a same row with different superscripts differ at *p* < 0.10.

1 SHAM, no castration with no treatment; CAS, castration with NaCl injection and administering oral lactose; LM, castration with lidocaine hydrochloride injection and administering oral meloxicam.

2 Standard error of mean.

Lying frequency was higher (*p* < 0.01) in CAS (17.1/4 h) than in SHAM (4.8/4 h), but it did not differ (*p* > 0.10) between CAS and LM (12.0/4 h). Lying duration/frequency had a treatment effect (*p* < 0.01), such that SHAM (19.5 min/no.) had the greatest values of this variable; there was no difference between CAS (3.8 min/no.) and LM (4.9 min/no.).

Walking frequency and duration were greater (*p* < 0.01) in both CAS (620/4 h and 51.5 min/4 h) and LM (529/4 h and 46.0 min/4 h) than in SHAM (209/4 h and 25.4 min/4 h). However, walking duration/frequency was greater (*p* = 0.04) in SHAM (0.13 min/no.) than in CAS (0.08 min/no.) and LM (0.09 min/no.).

All treatments differed (*p* < 0.01); the number of leg lifts and frequency of stiff gait were greatest in CAS (311/4 h and 93.9/4 h), intermediate in LM (159/4 h and 24.9/4 h), and least in SHAM (23.0/4 h and 1.3/4 h). The number of kicks was greater (*p* < 0.01) in CAS (83.3/4 h) than in SHAM (16.5/4 h) and LM (40.6/4 h).

Scratching frequency differed in all treatments (*p* < 0.01); the number was greatest in SHAM (49.8/4 h), followed by CAS (35.3/4 h) and LM (22.1/4 h).

## 4 Discussion

### 4.1 Growth performance

In this study, ADG tended to be lower in both castrated groups (CAS and LM) than in the SHAM group, whereas LM treatment did not improve growth performance. The negative effect of castration on growth has been reported (3, 6). The effect of LM treatment on growth is diverse. A study reported that the administration of meloxicam and lidocaine alone or in combination did not improve growth performance (14), but another study reported that meloxicam administration tended to increase the live weight, hot carcass weight, and overall ADG in castrated bulls (3). The reason for the inconsistency is unclear, but it may be due to differences in factors such as breed and animal age.

### 4.2 Plasma stress and inflammation indicators

Plasma cortisol is widely used as an indicator of distress in cattle (5). In this study, castration dramatically increased the cortisol level and lidocaine plus meloxicam treatment partially decreased the cortisol concentration at 0.5 h after castration. Our results are similar to those of (3, 6), who showed that the cortisol level increased in castrated calves compared with non-castrated calves at 0.5 h after castration. Lidocaine decreased the salivary cortisol at 30 min and 1 h after castration (14). The administration of meloxicam decreased the cortisol concentration from 2 to 5 h after surgical castration (25, 26). Collectively, these findings indicate that lidocaine plus meloxicam treatment is partially effective in alleviating the changes in cortisol concentration induced by acute stress, such as surgical castration.

In the present study, castration increased the haptoglobin concentration at 1 and 3 d after castration. LM treatment decreased the haptoglobin concentration compared with the untreated castrated group at 1 and 3 d after castration, but the haptoglobin concentration remained higher in the LM group than in the SHAM group, indicating that LM treatment caused partial reduction of the haptoglobin concentration. Haptoglobin is an acute phase protein that causes an anti-inflammatory response, which could serve as a marker of the extent of inflammation in cattle (27). Similar to our findings, previous studies showed that the haptoglobin concentration dramatically elevated from 6 h, peaked 3 d after surgical castration, then returned to the baseline level (6, 28). Our results were consistent with reports that meloxicam reduced the haptoglobin concentration at 1 to 4 d after surgical castration with oral administration (3, 28) and subcutaneous injection (14). Melendez et al. (14) suggested that administering a combination of lidocaine and meloxicam is more effective in terms of providing long-term alleviation of the pain and inflammation related to surgical castration, although no meloxicam × lidocaine interaction was observed. Collectively, these results indicate that lidocaine plus meloxicam treatment may be partially effective in alleviating inflammation for days after castration.

### 4.3 Heat shock protein 90 gene expression

Within the various HSP family, we focused on HSP 90, because it is a known biomarker of heat stress in cattle (29, 30) and has been studied in relation to animal pain (17, 18). In the present study, castration induced an increase in HSP90 mRNA at 0.5 and 6 h after castration, with levels returning to baseline after 1 d. As induced inflammation has been shown to increase HSP90 mRNA level in the rat (31, 32), this post-castration increase may be indicative of inflammation. Compared to plasma haptoglobin levels, which peaked at 3 d post-castration, HSP90 mRNA levels, which peaked at 1 d post-castration, appear to serve as a more rapid biomarker of inflammation. Furthermore, the administration of 17-dimethylaminoethylamino-17-demethoxygeldanamycin, an HSP90 inhibitor, could be considered as an alternative analgesic strategy for castration pain, as previously suggested (32, 33).

### 4.4 Behavior observations

Husbandry painful procedures such as castration and dehorning cause behavioral changes in cattle (9). In the present study, castration decreased the eating frequency and duration; LM treatment did not alleviate these changes in castrated bulls. Castration, dehorning, and concurrent castration and dehorning decreased the eating frequency (34). Similar to our findings, the administration of lidocaine and subcutaneous meloxicam alone or in combination did not increase the time spent eating (14).

In the present study, the CAS group drank more frequently and tended to drink longer than the SHAM and LM groups. In contrast, a previous study showed that untreated control calves spent the greatest length of time drinking compared with concurrently castrated and dehorned calves (9). This discrepancy may be due to the difference in observation time: the previous study collected 5 min focal samples for each animal at six different time points, whereas we observed each bull for 4 h continuously. Further research is needed to clarify the relationship among drinking behavior, castration, and LM treatment.

In the present study, castration caused the cattle to lie down less often and walk more. LM treatment did not alleviate these behaviors. Surgical castration causes calves to spend more time standing and walking and less time lying down (28, 35, 36). Previous research showed that the administration of meloxicam reduced the time spent walking, decreased the walking frequency (25, 35), and increased the duration of lying down (26, 36). According to Melendez et al. (14), the administration of lidocaine tended to reduce the duration of lying down. Our results indicated that castration makes bulls restless, and LM treatment was insufficient to mitigate the changes in lying and walking behaviors.

We found that castration increased the number of kicks and that LM treatment alleviated kicking frequency. These results are inconsistent with a previous study in which the administration of buccal meloxicam to 3-month-old ring-castrated beef calves did not alter the number of kicks (34). This discrepancy may be due to differences in castration method, observation method, and age. Notably, we used continuous sampling, rather than focal sampling, for behavior observations.

In the present study, castration increased the number of leg lifts and frequency of stiff gait, and LM treatment alleviated these changes. Here, we defined “leg lifting” behavior to include “stamping” and resemble “leg movement.” In previous reports, leg movement was greater among surgically castrated calves than among non-castrated calves at 2 to 4 h after castration (35, 36). Similarly, surgical castration increased the number of leg movements, which included leg lifting, in 6-month-old calves, whereas non-castrated calves had greater leg movement than surgically castrated calves in 3-month-old calves (23). Melendez et al. (35) suggested that the administration of subcutaneous meloxicam reduced the number of leg movements; in a subsequent study, Melendez et al. (36) found that the administration of subcutaneous meloxicam did not reduce the number of such movements. In previous research, the administration of lidocaine reduced the frequency of leg movement (14). The administration of a topical anesthetic and meloxicam alone or in combination reduced the frequency of stamping and stiff gait for 5 h after surgical castration (9). Overall, surgical castration induced leg lifting and stiff gait. Furthermore, LM treatment decreased the frequencies of leg lifting and stiff gait.

We found that the number of scratches was highest in the SHAM group, intermediate in the CAS group, and lowest in the LM group. A previous study did not detect an effect of castration on scratching behavior (9). Considering that the previous authors observed calf behavior for 5 min per hour, for a total of 25 min over 5 h, they presumably missed some scratching behavior, which might explain why they were unable to detect a castration effect. It is unclear why castrated bulls scratched their bodies less than control bulls, but castration-related pain may have been a distraction from the desire to scratch.

## 5 Conclusion

We found an increased HSP90 mRNA level in castrated calves, demonstrating the potential of leukocyte HSP90 mRNA level as a biomarker of inflammation. Surgical castration caused behavioral changes in eating, drinking, lying, walking, leg lifting, kicking, and stiff gait. The increased leg lift frequency may indicate that cattle were experiencing pain and discomfort. The partial reductions in the frequencies of lying, leg lifting, kicking, and stiff gait behaviors and the concentration of haptoglobin suggest that lidocaine plus meloxicam treatment is partially effective in terms of alleviating pain and inflammation in castrated bulls, improving animal welfare.

## Data Availability

The original contributions presented in the study are included in the article/Supplementary material, further inquiries can be directed to the corresponding author.
