# Supplementary material for: Effects of lidocaine-plus-meloxicam treatment on behavioral and physiological changes, and leukocyte heat shock protein 90 gene expression after surgical castration in Hanwoo bulls
Source: Front Vet Sci. 2024 Dec 4;11:1465844. doi: 10.3389/fvets.2024.1465844 (PMC11652710; doi:10.3389/fvets.2024.1465844)
Supplement: Supplementary file 1 [file Table_1.docx]

**Supplementary Table 1** Ingredients and chemical composition of diets for Hanwoo bulls.

| Ingredient or chemical composition | Percentage |
| --- | --- |
| Concentrate ingredients (DM basis) |  |
| Ground corn | 15.82 |
| Ground wheat | 18.00 |
| Salt | 0.88 |
| Molasses | 5.50 |
| Wheat bran | 3.00 |
| Corn flour | 5.00 |
| Rice bran | 3.00 |
| Cottonseed hulls | 1.50 |
| Palm kernel meal | 10.00 |
| Ammonium chloride | 0.15 |
| Rapeseed meal | 2.22 |
| Dried distilled grain solubles | 9.38 |
| Condensed molasses solubles | 1.50 |
| Corn gluten feed | 8.50 |
| Limestone | 3.30 |
| Copra meal | 10.00 |
| Porphyry | 2.00 |
| Vitamin/Mineral premix^1^ | 0.25 |
| Total | 100.0 |
| Concentrate composition |  |
| Dry matter | 88.60 |
| Crude ash | 7.56 |
| Crude protein | 18.03 |
| Crude fat | 4.40 |
| Acid detergent fiber | 15.64 |
| Neutral detergent fiber | 34.34 |
| Calcium | 1.22 |
| Phosphate | 0.65 |
| Total digestible nutrient (TDN)^2^ | 81.3 |
| Metabolizable energy^3^, MJ/kg | 12.0 |
| Timothy composition |  |
| Dry matter | 89.16 |
| Crude ash | 5.56 |
| Crude protein | 5.21 |
| Crude fat | 1.08 |
| Calcium | 0.16 |
| Phosphate | 0.16 |
| Acid detergent fiber | 32.33 |
| Neutral detergent fiber | 57.48 |
| Alfalfa composition |  |
| Dry matter | 89.14 |
| Crude ash | 6.43 |
| Crude protein | 9.11 |
| Crude fat | 0.85 |
| Calcium | 0.69 |
| Phosphate | 0.17 |
| Acid detergent fiber | 40.34 |
| Neutral detergent fiber | 52.80 |

^1^Vitamin and mineral premix contained 2,650,000 IU vitamin A, 530,000 IU vitamin D3, 1,050 IU vitamin E, 10 g Niacin, 4.4 g Mn, 4.4 g Zn, 13.2 g Fe, 2.2 g Cu, 0.44 g I, and 0.44 g Co per kg of additive (Grobic-DC, Bayer Health Care, Leverkusen, Germany).

^2^ TDN values of concentrate diets were provided from Cargill AgriPurina, Inc. feed company (Seongnam, Republic of Korea).

^3^ ME (MJ/kg) = 0.82 Ⅹ digestible energy^4^ (National Academies of Sciences, Engineering, and Medicine, 2016).

^4^ Digestible energy (MJ/kg) = 0.1845 Ⅹ total digestible nutrient (%) (National Academies of Sciences, Engineering, and Medicine, 2016).

| Gene name (Symbol) | Gene bank accession no. | Sequence (5'-3') | Tm,  °C | Length (bp) | Efficiency | R^2^ |
| --- | --- | --- | --- | --- | --- | --- |
| 18s ribosomal RNA (18S)^a^ | NR_036642.1 | For : TTTCGATGGTAGTCGCTGTGC Rev : TGCCTTCCTTGGATGTGGTAG | 61.0 59.7 | 108 | 98% | 0.99 |
| Heat shock protein 90 (HSP90) | NM_001012670.2 | For : GATCCCCAGACACATGCCAA Rev : TGGCATTTCCTCAGTGACCG | 60.0 60.3 | 114 | 97% | 0.98 |

**Supplementary Table 2** Sequences of the primers used in real-time PCR analyses.

^a^ 18S = Control gene

**Supplementary Table 3** Ethogram of behavior observed in this study

| Behavior | Definition |
| --- | --- |
| Eating | Head in the food trough |
| Drinking | Mouth around the waterer |
| Lying | Lying down completely on the ground in any style |
| Walking | Walking forwards or backwards in any style at any pace |
| Standing | Standing in any style |
| Leg lifting | Raising and lowering of front or hind foot (involve “stamp”) |
| Kicking | Kicking backward or towards the belly with a hind limb |
| Lesion licking | Number of times that the head was turned to lick the scrotal area |
| Head turn | Rapid turning of the head to either side of the body |
| Stiff gait | Walking slowly with muscles stiff |
| Scratch | Raising a hind leg and scratching part of the body or scratching body against the fence |
| Head shake | Rapid shaking of the head around a rostral to caudal axis |
| Head paw | Lifting hind leg and contacting the head |
